# Annotations

**Published:** 1908-03-14

**Authors:** 


### Annotations.

### The Mental Effects of Influenza.

Dr. F. S. Clouston, the retiring Physician Superintendent of the Royal Edinburgh Asylum for the Insane, in his final yearly report of the work of the institution during 1907 gave some interesting statistics of the cases which have come under his care. Dividing the Morningside mental cases into two large groups, the depressed or melancholic on the one hand, and the elevated and excited on the other, he finds that from 1874 to 1890 the number of maniacal patients was greatly in excess of the number of melancholics, the proportion for the 16 years being 60 per cent. to 40 per cent. Since 1890 the proportion of melancholic patients has increased to 48 per cent. for the period of eighteen years, and the two classes are now apparently about equal in number. 1890 was, of course, the year of the great epidemic of influenza, and Dr. Clouston's suggestion is that the increasing proportion of melancholic cases is attributable to influenza. This argument, like many others dependent on statistics, is open to the objection that is is of a *post hoc* character, but it is none the less interesting for that reason. Among other allied after-effects of influenza, Dr. Clouston mentions neurasthenic conditions, premature senility, various forms of paralysis, neuralgic affections of every kind, and a general incapacity for work, both bodily and mental. He further says of influenza, and there are many who will agree with him, that "no disease of whose effect there is any correct record has had such far-reaching evil effects." Dr. Clouston's suggestion of the strong, if remote, causal relationship between influenza and melancholia comes at an appropriate time, and deserves careful attention.

### Pot Still and Patent Still.

At the second and third public sittings of the Royal Commission on Whiskey and Other Potable Spirits evidence was given by Mr. Aaron Richardson, Inspector of Customs; Mr. A. M. Bramall, solicitor to the Islington Borough Council; Mr. A. J. Walter, K.C.; and Dr. W. Murrell, physician to Westminster Hospital. Mr. Richardson gave information concerning spirits imported from abroad. He said that there was no legal definition of brandy any more than there was a legal definition of whiskey. Mr. Bramall urged that the public should have full knowledge by label or otherwise of the nature, character, and age of the spirit which they bought. Whiskey, in his opinion, should be made from malt and unmalted barley, with small additions of other materials, and it should be made in pot-stills; while Scotch and Irish whiskeys should be made in their respective countries. According to his view no patent-still product could properly claim the title of whiskey. He said that many of the pot-still distillers were terrorised by the blenders, in whose hands they were. He condemned the widespread practice of concocting whiskey from patent-still spirit flavoured with essences. Mr. Walter, K.C., defended the product of the patent-still, maintaining that it was as much entitled to the definition "whiskey" as the pot-still spirit, and that the public taste was tending towards a purer alcohol. He pointed out the difficulties in the way of stating upon the label the proportion of the constituent spirits in a blended whiskey. Dr. Murrell said that the beneficial effects of whiskey in the treatment of acute pneumonia, enteric fever, influenza, and other specific fevers were due not so much to the alcohol as to the by-products, especially the ethers. In debilitated conditions generally, whether cardiac, gastric, or circulatory, especially in elderly people, the therapeutic value of whiskey, according to Dr. Murrell, is again derived from the by-products more than from the alcohol. Rectified spirit, in his experience, produces no corresponding benefit in such cases. These by-products increase and develop with age: hence the improvement of pot-still whiskey with age. Dr. Murrell does not think that patent-still spirit deserves the name of whiskey at all.

### Expert Medical Evidence in America.

The degraded state into which expert medical testimony has sunk in America is recognised and deplored throughout that country. The educated public, as well as the legal and medical professions, are anxiously inquiring into the causes of the evil, with a view to the discovery of a remedy. Dr. A. E. Osborne, in the *California State Journal of Medicine*, reviews the situation and quotes the opinions of medical men, lawyers, and laymen. As is to be expected, there is some diversity of view, though all are agreed that sweeping reform is urgently needed. Several authorities believe that the fault lies in the system, the law and the principles of evidence being to a large extent responsible for the present state of affairs. A medical writer attributes much of the evil to the impossibility of counsel understanding a difficult medical situation or framing questions calculated to bring out the point at issue. Another and more pessimistic physician believes that no matter how perfect the expert evidence given the verdict would not be influenced one whit so long as jurymen are entrusted with the determination of questions so far beyond their comprehension. Dr. Osborne utterly condemns the "hypothetical question," so much beloved of cross-examining counsel: this form of questioning an expert should, he maintains, be relegated to a museum for antiques. This, together with the practice of retaining paid expert witnesses on one side or the other, must be abolished. The medical expert of the future must have genuine claims to that title; he must be selected by the court and paid by the court; and he must be given opportunity to render a full and unhampered professional opinion based upon ascertainable facts. Such are Dr. Osborne's proposals for reform. In time, perhaps, if this were done, the public of the United States would cease to accept as a fact that the paid medical expert swears for the side that engages and compensates him.

